# Integrative Application of Foliar Yeast Extract and Gibberellic Acid Improves Morpho-Physiological Responses and Nutrient Uptake of *Solidago virgaurea* Plant in Alkaline Soil

**DOI:** 10.3390/life12091405

**Published:** 2022-09-09

**Authors:** Samah M. Youssef, Ebtsam M. M. Abdella, Omar A. Al-Elwany, Khalid S. Alshallash, Khadiga Alharbi, Mariam T. S. Ibrahim, Moataz M. Tawfik, Abdelghafar M. Abu-Elsaoud, Amr Elkelish

**Affiliations:** 1 Horticulture Department, Faculty of Agriculture, Fayoum University, Fayoum 63514, Egypt; 2 College of Science and Humanities—Huraymila, Imam Mohammed Bin Saud Islamic University (IMSIU), Riyadh 11432, Saudi Arabia; 3 Department of Biology, College of Science, Princess Nourah bint Abdulrahman University, P.O. Box 84428, Riyadh 11671, Saudi Arabia; 4 Department of Biochemistry, Faculty of Agriculture, Ain Shams University, Cairo 11566, Egypt; 5 Botany Department, Faculty of Science, Port Said University, Port Said 42526, Egypt; 6 Department of Botany and Microbiology, Faculty of Science, Suez Canal University, Ismailia 41522, Egypt

**Keywords:** solidago, *Solidago virgaurea*, *Asteraceae*, alkali stress, foliar application, active dry yeast, gibberellins

## Abstract

Alkaline soils have fertility issues due to poor physical qualities, which have a negative impact on crop growth and output. Solidago is used in flower arrangements, bouquet filler, and traditional medicine. The possible biological fertilizers’ eco-friendly and cost-effective nature favours farmers because of the vital role in soil productivity and environmental sustainability. A field experiment was performed during two successive seasons to explore the effect of applying yeast extract (YE) at (0, 0.5, 1.0, and 1.5 g/L) and/or gibberellic acid (GA_3_) at (control, 100, 200, and 300 ppm) on the morpho-physiological parameters, macronutrients, and biochemical constituents of *Solidago virgaurea*. The results emphasize that YE (1.5 g/L) and/or GA_3_ (300 ppm) treatments show the highest significant increase in plant growth (i.e., plant height, no. of branches, fresh and dry weight of shoots); photosynthetic efficiency (i.e., chlorophyll (a), chlorophyll (b) and total carotenoids); macronutrient content (i.e., N, P, and K); and biochemical constituents (i.e., total soluble sugars, total phenolic, total flavonoids, and total glycosides). The study results recommend using YE and GA_3_ in combination at concentrations of 1.5 g/L and 300 ppm, respectively, to improve Solidago production sustainability under alkaline soil conditions.

## 1. Introduction

Solidago, often known as golden rod, is a member of the *Asteraceae* family. There are roughly 130 species in this genus, most of which are found in North America [1]. *Solidago virgaurea*, *S. canadensis*, and *S. memoralis* are species grown in beds, borders, and rock gardens. They are widely utilized as cut flowers for bouquets and indoor decorating [2,3]. One of the most important species in the Solidago genus is the European goldenrod (*Solidago virgaurea* L.) plant. It is grown as a perennial flowering plant that is prized for its tall, straight flower stalks [3,4]. The aerial parts of Solidago have been used in traditional medicine for millennia as anti-inflammatory, spasmolytic, and diuretic remedies for a variety of ailments, particularly as a urological agent in kidney and bladder inflammation, urolithiasis, and cystitis, and a yellow dye was produced from the flowers [4,5,6]. Furthermore, it is a source of several significant secondary metabolites, all of which have great therapeutic potential. Moreover, Solidago has been shown to have anticancer activity on human prostate (PC-3), breast (MDA435), melanoma (C8161), and small cell lung carcinomas (H520) [6]. According to the European Medicines Agency, *S. virgaurea* is one of the most investigated and used species of the Solidago genus in Europe [3]. Flavonoids (mainly quercetin glycosides); salicylic acid derivatives (leiocarposide, virgaureoside); caffeoylquinic acid derivatives (chlorogenic acid, caffeic acid); triterpene saponins (oleanane type); tannins; and essential oils are all found in *S. virgaurea* extracts [7]. Several bioactive chemicals found in *S. virgaurea* extracts work together: flavonoids, saponins, caffeic acid derivatives, and leiocarposide have anti-inflammatory properties, polyphenolic compounds have antioxidant properties, and flavonoids have spasmolytic properties [8,9,10]. Soil plays a crucial part in defining an agro-long-term ecosystem’s productivity. The ability of soil to provide critical nutrients to growing plants determines its sustainable productivity. Micronutrient insufficiency has become a key barrier to soil productivity, stability, and sustainability [9]. Soil alkalinity is one of the most common concerns in arid and semi-arid areas, with pH levels ranging from 7.5 to 8.7 [11,12,13]. Arid land makes up 97% of Egypt’s total land area, and it is characterized by high temperatures, low relative humidity, rapid evaporation, and minimal rainfall, resulting in degraded soils [12]. Soil alkalinization induced by NaHCO_3_ and Na_2_CO_3_ may be more serious than soil salinization caused by neutral salts such as NaCl and Na_2_SO_4_ [14,15,16]. Egypt’s soils are characterized by pH values that range from slightly alkaline to alkaline, owing to the country’s arid climate. As soil salinization and alkalinization frequently co-occur and are quite complicated, total salt concentration, composition, and the proportion of neutral to alkaline salts can all differ significantly between soils [15]. Furthermore, alkali stress is characterized by a mixture of stresses, osmotic, ion-induced damage, and a high pH level [12]. Alkalinity interferes with the uptake of nutrients that contribute to growth and the accumulation of bioactive components. Foliar nutrition is one of the most effective agricultural practices for health growth. Foliar application can also provide 85% of the plant nutritional requirements [16]. Otherwise, there has recently been a worldwide movement toward using natural compounds that are both safe and non-polluting to the environment.

In particular, the plant’s capacity to produce primary and secondary metabolites is significantly influenced by soil conditions [17]. Prior research showed that some chemical features of soil are very unique to certain plant species [18]. Klimien and his co-worker [19] found that oregano grown in acidic soil reaction led to a lower content of total phenols and extractives in the raw material compared to an alkaline soil.

Active dry yeast (YE) is a natural and safe biofertilizer that plays an important function in plant growth. It is a rich source of essential nutrients, particularly cytokinins, which operate as a rapidly available growth supplement for plants, resulting in increased yield [20]. As a result, it aids in cell division and expansion, protein and nucleic acid synthesis, and chlorophyll creation, all of which contribute to improved plant growth [21]. It is also high in amino acids, peptides, and B-complex vitamins, including B1, B2, B6, and B12, as well as carbohydrates, sugars, and minerals. It contains several amino acids, vitamins, and essential elements such as Na, Ca, Fe, K, P, S, Mg, Zn, and Si [22]. It also emits CO_2_, resulting in an improvement in net photosynthesis [23]. In marigold plants, using yeast as a foliar fertilizer improved growth and plant nutrition [24]. In the white lupine, when compared to untreated plants, the varied active yeast extract treatments significantly improved the growth and physiological properties [25]. Internal variables such as hormonal and nutritional balance regulate plant growth and development. Growth regulators, which are increasingly being utilized to influence the growth and flowering of ornamental and medicinal plants, are responsible for the balanced development of plants [26]. At optimal concentrations, plant growth regulators are known to coordinate and control several phases of growth and development, including flowering. The exogenous application of plant growth regulators alters the concentrations of naturally existing hormones, which then affects the plant’s growth and development [27]. Gibberellins (GAs) are found in both flowering and non-flowering plants, and they are widely distributed. GAs, especially gibberellic acid (GA_3_), belong to the diterpenoid class of bioactive growth regulators [28]. GA_3_ has the ability to alter the growth pattern of plants by influencing DNA and RNA levels, cell division and expansion, enzyme production, protein, carbohydrate biosynthesis, and photosynthetic pigment biosynthesis [1]. Different GA_3_ concentrations substantially affected all golden rod growth, flower quality, and yield characteristics [29]. The application of GA_3_ at various doses to marigold plants positively influenced many growth parameters, flower quality, and yield attributes [30].

To date, no research has been conducted on the effects of yeast extract alone or in combination with gibberellic acid on European goldenrod. Therefore, in light of the previous facts, the current investigation was implemented to assess the advantages of YE and/or GA_3_ amendments and their reflections on morpho-physiological characteristics, nutritional accumulation, and biochemical determinations of Solidago.

## 2. Materials and Methods

### 2.1. Experimental Layout and Soil Analysis

The experiments took place in an open private field (29°19′23.2″ N 30°51′27.5″ E) located in Fayoum Governorate, Egypt. The two seasons began in March 2018 and ended in July of the same year, and this was repeated over the same period in 2019. Before each experiment, soil samples from the experimental site were collected and evaluated. The physical and chemical parameters of the tested soil were determined using some conventional published methodologies [30,31], and the results are summarized in Table 1 The analysed data of the tested soil revealed that it has a high pH value (7.82), indicating that it is classified as it tends to alkalinity. Therefore, this soil tends to fix many essential nutrients, especially P, Fe, Zn, Cu, Mn, and B [12].

### 2.2. Plant Material

Standardized Solidago seedlings, (length 5 cm, 2–3 pairs of true extended leaves) were purchased from the Agricultural Research Centre, Giza, Egypt. On 5 March, the seedlings were transplanted in plots of 4 m in length and a 2.5 m width. Each plot had three rows, at a pace of two seedlings per hole, and approximately 50 cm between the seedlings within rows.

### 2.3. Treatments and Experimental Design

The experimental arrangement was a factorial experiment based on a randomized complete block design (RCBD) with three replications. We started the experiment with a large number of treatments. The preliminary study included yeast extract concentrations at (0.5, 1.0, 1.5, 2.0 and 2.5 g**/**L) and gibberellic acid concentrations at (100, 200, 300, 400 and 500 ppm). According to the results of this study, we found that the levels 1.5 g/L for yeast extract and 300 ppm for gibberellic acid exhibited the best plant performance. The higher levels (2, 2.5 g/L for yeast and 400, 500 ppm for gibberellic acid) did not show any significant improvements. Then, 16 treatments were started, as follows: yeast extract (YE) in four different concentrations, which correspond to zero as control, 0.5, 1.0, and 1.5 g/L, and gibberellic acid (GA_3_) (control, 100, 200, and 300 ppm). The plants in the control group were foliar sprayed with distilled water. The foliar spray application of YE was applied 30 days after transplantation, and the second spray was applied 15 days later. Following two days of YE treatment, the plants were treated with GA_3_. The active dry yeast powder was acquired from a local market. Sucrose was used to activate yeast overnight. The yeast extract was conducted to the chemical analysis for knowing its components [32,33], as shown in Table 2. During the seasonal growth period, all the experimental units received equal dosages of Karma NPK fertilizer (Sphinx for International Trading Co., Nasr city, Egypt) at a rate of 300 gm^−2^ twice.

### 2.4. Agricultural Practices

Standard agronomic procedures such as weeding, irrigation, and pest control were achieved as needed and suggested in the commercial production of European goldenrod.

### 2.5. Recording Data

#### 2.5.1. Morphological Measurements

After 85 days of transplanting, five randomly selected plants from each experimental unit were cut off at the ground level and transported to the laboratory. Plant height (cm). The plant’s height was measured from the ground to its tip, and the average was calculated and given in cm, alongside the number of branches/plant, and the fresh and dry weight of the shoots (g plant^−1^). Each plant’s shoots were dried in an electric oven at 70 °C until they attained a constant weight.

All chemicals used in the analyses were purchased from Sigma—Munich, Germany.

#### 2.5.2. Physiological Determinations

Chlorophyll a, b, and total carotenoids (mg/mm^2^) were calculated by utilizing the dimethyl formamide (DMF) method [33,34]. Using a spectrophotometer 160A UV-Visible (Shimadzu Company, Kyoto, Japan), the absorption at wave lengths of 647 nm and 664 nm was measured to determine the amounts of chlorophyll a and b. The calculations below were used to compute the concentrations of chlorophyll a and b [34]:

Chlorophyll a (Chl a) = 11.65 A_664_ − 2.69 A_647_        (x)

Chlorophyll b (Chl b) = 20.81 A_647_ − 4.53 A_664_        (xi)

The following calculations were used to determine the chlorophyll a and b contents (g mm^−2^) based on the sample disc area and the measured chlorophyll a and b concentrations:

Chlorophyll a (µg mm^−2^) = (x)/disc area

Chlorophyll b (µg mm^−2^) = (xi)/disc area

Total Chlorophyll = Chl a + Chl b

The total carotenoids content was assessed by the absorption at wave length 480 nm via a spectrophotometer (UV-Visible Spectroscopy System, Hewlett Packard 95–98). The following formula was used to calculate the concentration of total carotenoids:

Total carotenoids (car) = [1000 A_480_ − 0.89 (Chl a) − 52.02 (Chl b)]/245    (xii)

From the sample disc area and the measured total carotenoid concentration, the total carotenoids content (µg mm^−2^) was calculated using the following formula:

Total carotenoids (µg mm^−2^) = (xii)/disc area

#### 2.5.3. Macronutrients Determinations

The Orange G dye was used to colorimetrically evaluate the nitrogen content of the leaves [34]. To make an Orange-G dye solution, 1.0 g of 96% (*w*/*w*) test dye was dissolved in 1.0 L of distilled water along with 21.0 g of citric acid, which served as a buffer to maintain the proper pH, and 2.5 mL of 10% (*v*/*v*) thymol in alcohol, which served as a microbial growth inhibitor. A centrifuge tube was filled with 0.2 g of ground plant leaf material and 20 mL of the dye reagent solution. The tube’s contents were shaken on an auto-shaker for 15 min at 300 rpm. Following filtering, the solution was diluted 100 times with distilled water, and the absorbance of the mixture was determined at 482 nm. The following formulas were used to calculate the N contents: N% = 0.39 + 0.954 × Dye absorbed (g/100 g) and Dye absorbed (g/100 g) = $\frac{a-b}{a}\times \frac{\mathrm{cfv}}{w}\times 100$.

Where a represents the absorbance of the dye reagent solution at 482 nm without any plant material (blank); b represents the absorbance of the dye reagent solution at 482 nm with plant material; c reflects the dye reagent’s concentration (1 g/L of distilled water); f represents the dye reagent’s purity percentage (96%); v represents the volume of the dye reagent solution used per sample (20 mL); and w is the dry material’s weight in g (0.2).

For phosphorus measurements in leaf tissue, the molybdenum-reduced molybdophosphoric blue colour method in sulphuric acid was utilised [35]. Sulphomolybdic acid was also diluted, and the reagents sodium bisulphite-H_2_SO_4_ solution 8% (*w*/*v*) were utilised.

Potassium was estimated using a flame photometer (Gallenkamp Company, London, UK) Perkin–Elmer model 52 with acetylene burner. A flame photometer works by atomizing a solution sample into a flame, which separates the characteristic spectra of an element and measures the emission at the same time. Flame-based devices with operating temperatures between 1000 and 3000 °C produce little excitation [36].

#### 2.5.4. Biochemical Constituents

Total soluble sugars were measured colorimetrically using dry matter from the fully expanded leaves of five plants selected at 85 days following transplanting, according to published methods [37]. Fresh leaf samples weighing 0.2 g were homogenized in 10 mL of 96% (*v*/*v*) ethanol before being rinsed in 5 mL of 70% (*v*/*v*) ethanol. The supernatant from the centrifugation of the extract at 3500 *g* for 10 min was kept at 4 °C for measurement. By combining 0.1 mL of the ethanolic extract with 3 mL of freshly made anthrone reagent (150 mg of anthrone with 100 mL of 72% (*v*/*v*) sulphuric acid) and heating the mixture in a boiling water bath for 10 min, it was possible to measure the concentrations of total soluble sugars. After cooling, a Bausch and Lomb-2000 Spectronic Spectrophotometer (Vaughan, ON, Canada) was used to measure the mixture’s absorbance at 625 nm.

The Folin–Ciocalteu colorimetric technique was used to evaluate the total phenolic content using the same methanolic extract of dry material [38]. In brief, 0.1 g of leaf powder was dissolved in 1 mL of deionized water. A total of 0.1 mL of this solution, 2.8 mL of deionized water, 2 mL of sodium carbonate (Na_2_CO_3_), and 0.1 mL of the 50% Folin–Ciocalteau reagent were combined. The reaction mixture’s absorbance was measured at 750 nm against a deionized water blank on a spectrophotometer 160A UV-Visible (Shimadzu Company, Kyoto, Japan) following a 30-min incubation at room temperature.

The quantities of flavonoids were measured using a colorimetric test technique [39], with minor modifications published before [40]. To establish linear calibration with function, rutin was utilized as a standard: A = 8.0045 C + 0.0914; r = 0.9959 (r = linear range). The total flavonoid concentration was measured in mg of rutin equivalent per g of dry weight (DW). In brief, 0.1 g of leaf was dissolved in 1 mL of deionized water. This solution (0.5 mL) was combined with 1.5 mL of 95% alcohol, 0.1 mL of 10% aluminium chloride hexahydrate (AlCl_3_), 0.1 mL of 1 M potassium acetate (CH_3_COOK), and 2.8 mL of deionized water. The reaction mixture’s absorbance was measured at 415 nm against a blank of deionized water using a spectrophotometer, following a 40-min incubation at room temperature.

The total glycosides were quantified using process [41], and the details of the modification and standard curve were explained [42]. A 10% extract was briefly combined with 10 mL of freshly made Baljet reagent (95 mL of 1% picric acid + 5 mL of 10% NaOH). The mixture was diluted with 20 mL of distilled water after an hour, and a spectrophotometer was used to determine the absorbance at 495 nm. Total glycosides were expressed as mg of securidaside per g of dried extracts from triple repetitions.

### 2.6. Statistical Analysis

The collected data were statistically analysed according to an analysis of variance for factorial design by InfoStat computer software package (version 2012) (Córdoba, Argentina). A two-way analysis of variance was used to examine the data (ANOVA). Duncan’s multiple ranges as a post hoc test was used to analyse the differences between treatment means at *p* ≤ 0.05 [43].

## 3. Results

### 3.1. Effects of YE and GA_3_ Spraying on the Morphological Measurements of the Solidago Virgaurea Plant, as Well as Their Interactions

**Figure 1:**
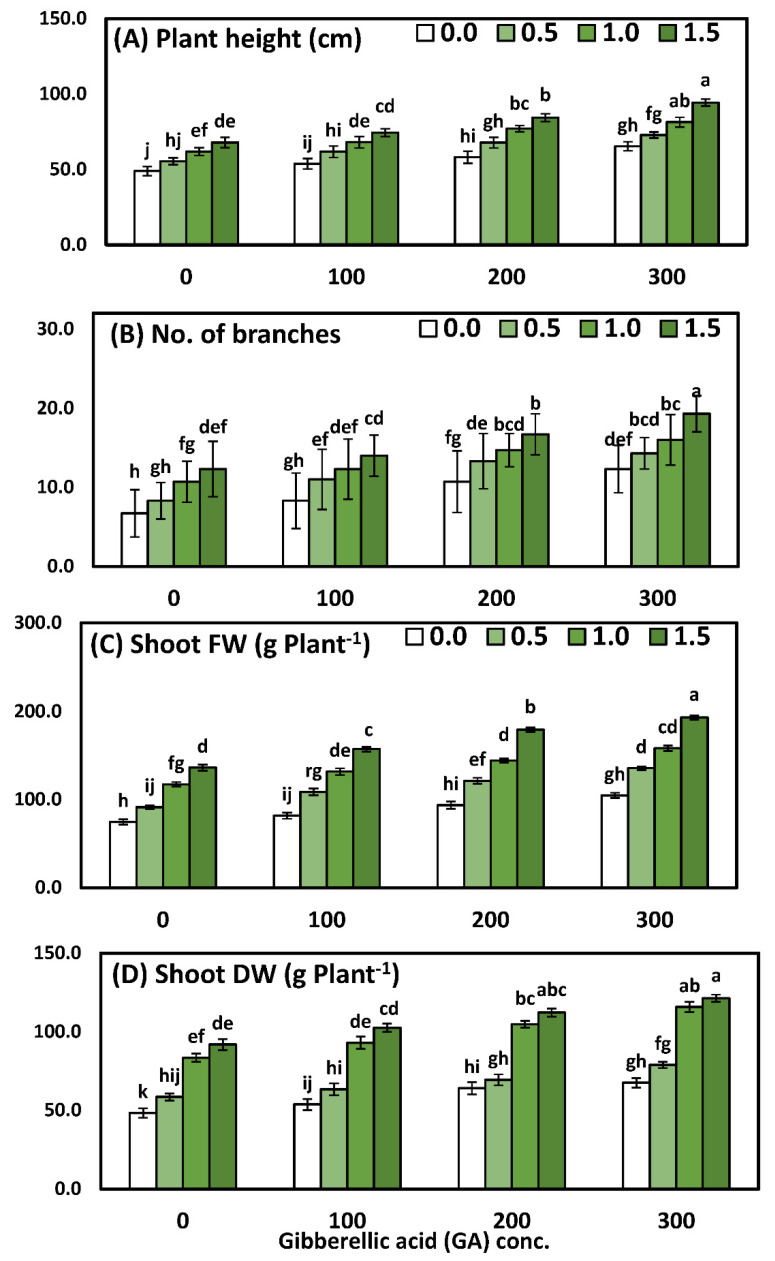
The interactive effect of yeast extract (0, 0.5, 1, and 1.5 g/L) and gibberellic acid (0, 100, 200, and 300 ppm) on the morphological measurements (*Solidago virgaurea*) of plants. (**A**) Plant height, (**B**) number of branches, (**C**) shoot fresh weight, (**D**) shoot dry weight. Bars with different letters are significantly different according to DMRTs at 0.05 level.

Solidago morphological measurements, namely plant height, the number of branches, the fresh and dry weight of shoots responses YE, and the GA_3_ concentrations and interaction of foliar application YE with GA3 concentrations, are illustrated in Table 3 and Figure 1. Using a two-way analysis of variance, a highly significant difference was seen in the morphological parameters in response to different yeast concentrations (*p* < 0.001), GA concentrations (*p* < 0.001), and the interaction between YE and GA treatments (*p* < 0.001).

Comparisons among the four concentrations of YE indicated that all the growth parameters increased significantly and progressively with every increment of the YE concentrations. Likewise, the application of the highest concentration of YE (1.5 g/L) was pioneering and recorded significantly higher mean values of plant height 80.2 cm, the no. of branches 15.6/plant, shoot fresh weight 166.6 g, and shoot dry weight 107.0 g, compared to the plants that were sprayed with distilled water. With regard to the effect of GA_3_, the results show that all GA_3_ concentrations caused significant enhancement for all growth parameters. Furthermore, the highest values of plant height 78.4 cm, the no. of branches 15.5/plant, shoot fresh weight 148.0 g, and shoot dry weight 95.9 g were detected by using GA_3_ at 300 ppm, in comparison to the control group, which achieved the lowest results. Comparisons among the sixteen mean values of the interaction between the two studied factors had a significant effect on all the growth parameters, as compared to the control or each one alone. Furthermore, the application of YE at 1.5 g/L together with 300 ppm GA_3_ significantly achieved the highest values, which were plant height 94.3 cm, the no. of branches 19.3/plant, shoot fresh weight 193.3 g, and shoot dry weight 121.3 g.

### 3.2. Effects of YE and GA_3_ Spraying on the Physiological Measurements of the Solidago Virgaurea Plant, as Well as Their Interactions

**Figure 2:**
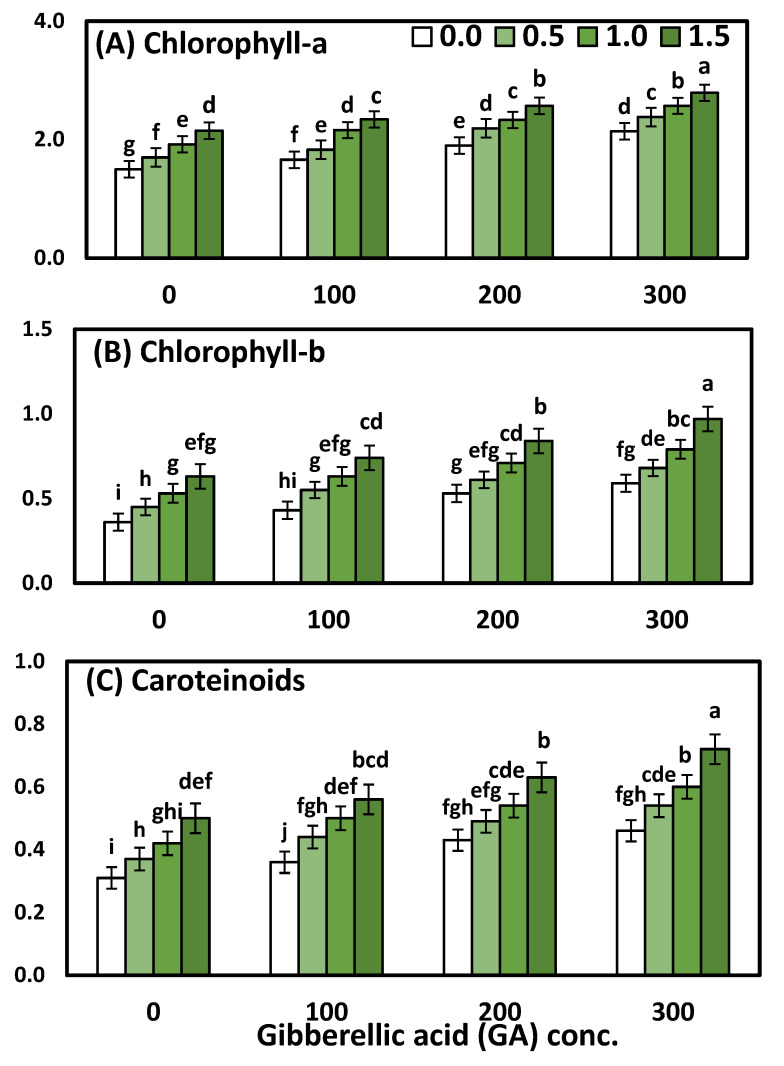
The effect of YE (0, 0.5, 1, and 1.5 g/L) and GA_3_ (0, 100, 200, and 300 ppm) on (**A**) chlorophyll (a), (**B**) chlorophyll (b) and (**C**) carotenoids of *Solidago virgaurea* plants. Bars with different letters are significantly different according to DMRTs at 0.05 level.

Chlorophyll (a), chlorophyll (b), and total carotenoids were affected significantly (*p* < 0.001 ***) by YE, the GA_3_ concentrations, as well as their interactions, which are presented in Table 4 and Figure 2. The two-way analysis of variance revealed a highly significant difference in the physiological parameters (chl-a, chl-b, carotenoids) in response to different YE concentrations (*p* < 0.001), GA concentrations (*p* < 0.001), and the interaction between the YE and GA treatments (*p* < 0.001).

Regarding the effect of YE, the data showed that the general influence of YE spray at different concentrations on all chl-a, chl-ba, and carotenoids was significant. Likewise, the mean values of chlorophyll (a), chlorophyll (b), and total carotenoids were in ascending order, as the YE concentration increased up to the highest concentration. Concerning the GA_3_ concentrations, it is clear that the highest one (300 ppm) of GA_3_ excelled the rest of the concentrations, which were markedly increased in all physiological parameters compared to the plants that were sprayed with distilled water and other concentrations of GA_3_. Furthermore, the increment reached 35.71, 55.10 and 45%, chlorophyll (a) chlorophyll (b), and total carotenoids, respectively. A significant interaction effect between the two studied factors on all the physiological parameters was evident in both seasons. Otherwise, the treatment combination of YE at 1.5 g/L with GA_3_ at 300 ppm gave the highest significant chlorophyll (a) 2.79 mg mm^−2^, chlorophyll (b) 0.97 mg mm^−2^, and total carotenoids 0.72 mg mm^−2^, as compared to the control and each one alone.

### 3.3. Effects of YE and GA_3_ Spraying on the Macronutrient’s Measurements of the Solidago Virgaurea Plant, as Well as Their Interactions

**Figure 3:**
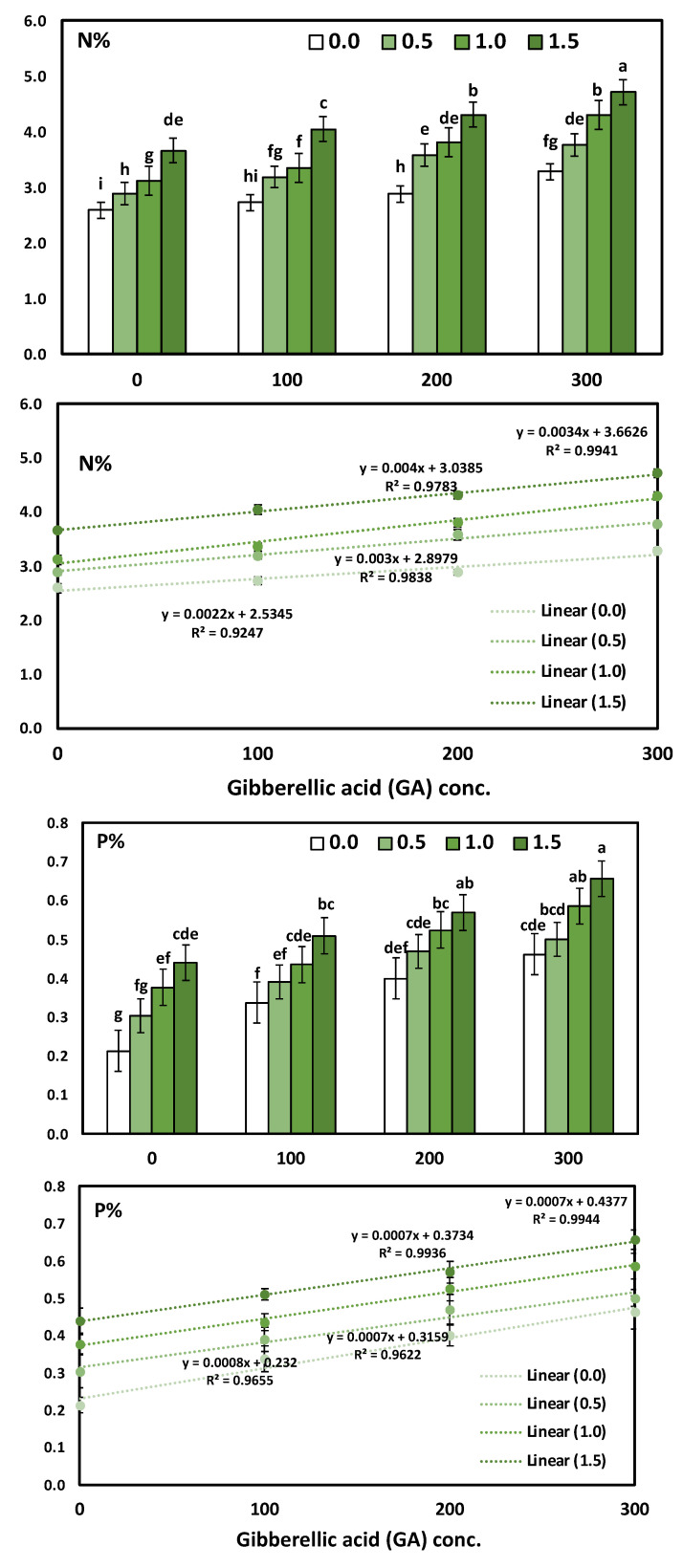

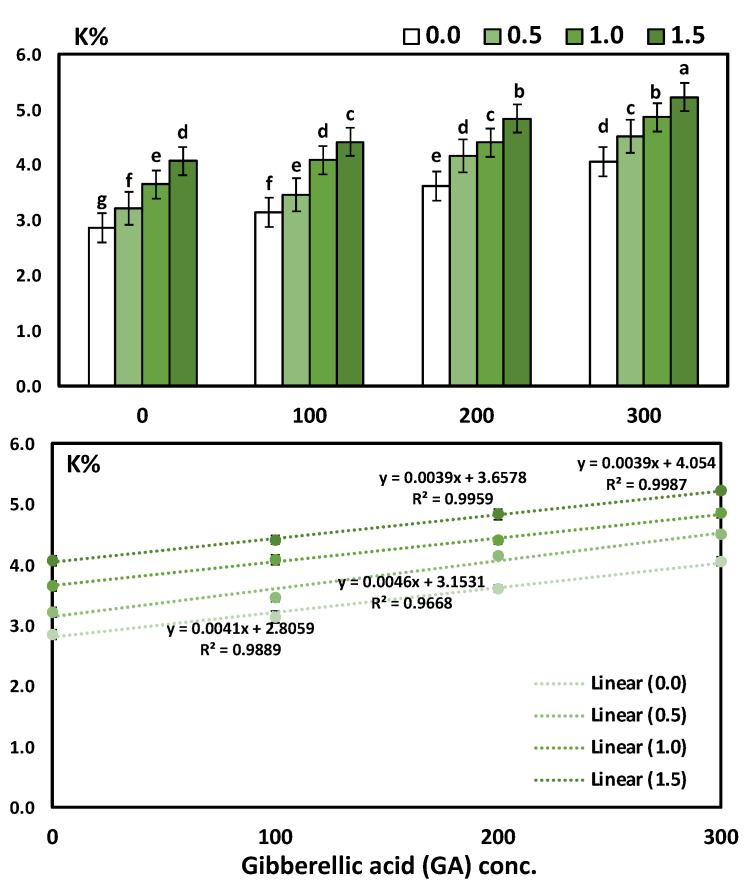
The interaction effect of yeast extract and gibberellic acid on the macronutrients constitute (*Solidago virgaurea*) plants. Bars followed by different letters are significantly different according to DMRTs at 0.05 level.

Table 5 and Figure 3 shows N, P, and K in response to YE, the GA_3_ concentrations, and their interactions. The two-way analysis of variance revealed a highly significant difference in macronutrients (N, P, and K) in response to different yeast concentrations (*p* < 0.001), GA concentrations (*p* < 0.001), and the interaction between the YE and GA treatments (*p* < 0.001).

The main effect of YE applied at different concentrations on the accumulation of the content N, P, and K % in the leaves showed a positive correlation. Likewise, the application of YE irrespective to the concentrations used significantly increased the leaves’ N, P and K %, as compared to the control treatment. Moreover, the application of YE at a high concentration (1.5 g/L) showed a highly significant increase in the leaves’ N (4.18%), P (0.54%), and K (4.64%). As for the effect of GA_3_, the results indicate that all the GA_3_ concentrations significantly augmented in the N, P, and K % content in the leaves compared to the control treatment. Furthermore, the increases in the mean values of N, P, and K % were linearly correlated with the increase in GA_3_. The treatment combinations of YE and GA_3_ concentrations seemed to have a more significant effect on the N, P, and K % in the leaves. Otherwise, the highest results were gained in the content of N, P, and K % in the leaves due to using YE at 1.5 g/L together with GA_3_ at 300 ppm in the two growing seasons, as compared to the control treatment. The above-mentioned N, P, and K % enhanced by 81.85%, 214.29%, and 82.52%, respectively.

The GA_3_ concentration significantly induced a significant positive increase in N, P, and K, as revealed by Pearson’s correlation and simple linear regression. The determination coefficient R^2^ ranged from 0.924 to 0.9941, which indicates a strong positive increase in the GA concentrations. The regression trendline was presented in Figure 3 for N, P, and K with the simple linear regression equation and determination coefficient (R^2^).

### 3.4. Effects of YE and GA_3_ Spraying on the Total Soluble Sugars, Total Phenolic, Total Flavonoids and Total Glycosides Measurements of the Solidago Virgaurea Plant, as Well as Their Interactions

**Figure 4:**
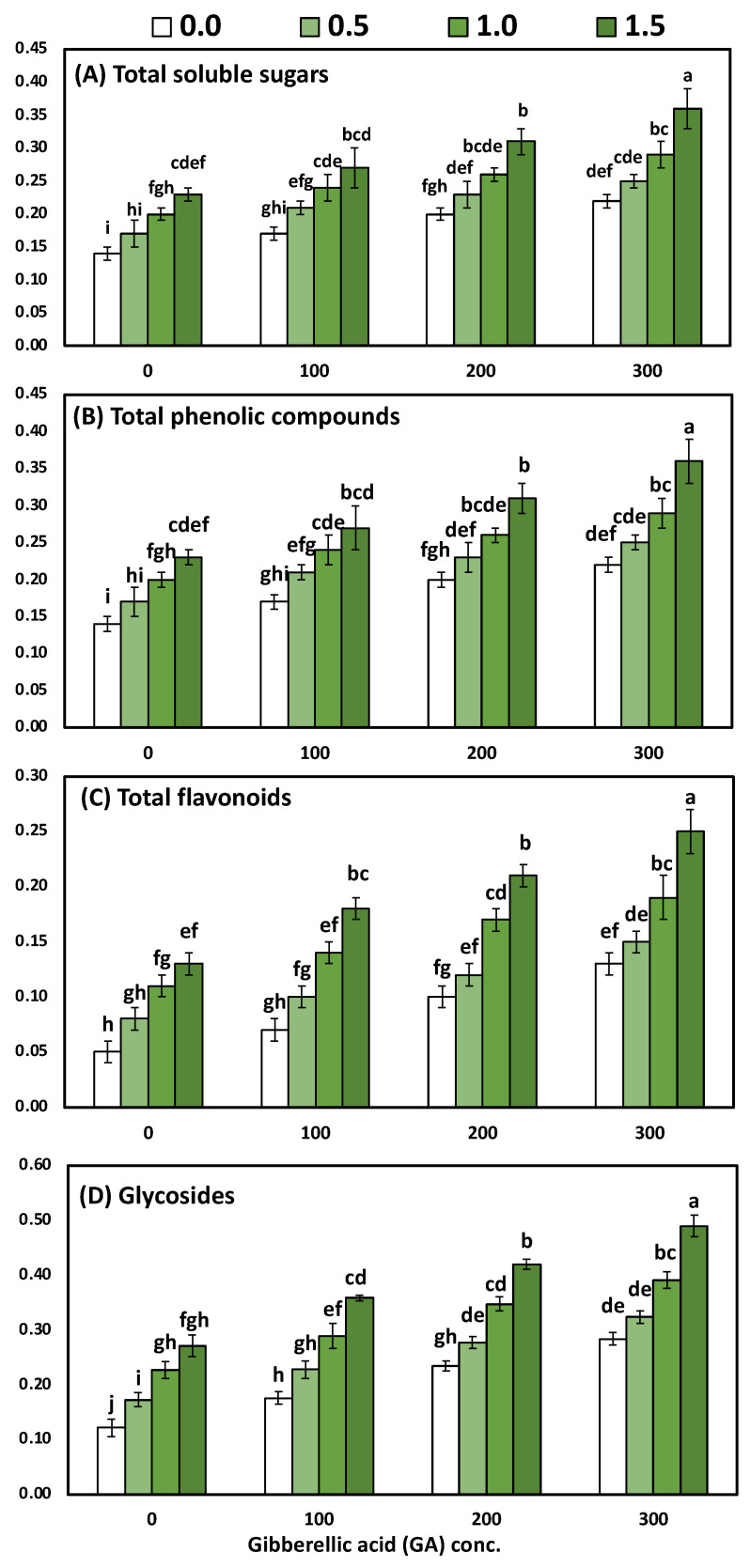
The interaction effect of yeast extract and gibberellic acid on the biochemical constituents of (*Solidago virgaurea*) plants including (**A**) total soluble sugars (mg/g-DW); (**B**) total phenolic compounds (mg/g-DW); (**C**) total flavonoids (mg/g-DW); and (**D**) total glycosides (mg/g-DW). Bars with different letters are significantly different according to DMRTs at 0.05 level.

Table 6 and Figure 4 display the main effects of the two studied factors (YE and GA_3_ concentrations) and their interactions on total soluble sugars, total phenolic, total flavonoids and total glycosides in leaves. The two-way analysis of variance revealed a highly significant difference in the biochemical parameters (total soluble sugars, total flavonoids, total phenolic compounds) in response to different yeast concentrations (*p* < 0.001), GA concentrations (*p* < 0.001), and the interaction between YE and GA treatments (*p* < 0.001).

The differences between the four concentrations of YE on the leaves’ total soluble sugars, total phenolic, total flavonoids, and total glycosides were significant. Likewise, progressive increases in all the biochemical constituents occurred due to the foliar application of YE up to the highest concentration, whereas the highest values (0.29, 0.31, 0.20 and 0.38 mg/g DW, total soluble sugars, total phenolic, total flavonoids, and total glycosides, respectively) were detected by 1.5 g/L with YE in both seasons. The detected differences among the mean values of total soluble sugars, total phenolic, total flavonoids, and total glycosides in the leaves within the four utilized concentrations of GA_3_ were enough to be significant. Likewise, all the biochemical constituents in the leaves were positively correlated with increasing GA_3_ concentrations, whereas the 300-ppm treatment occupied the first rank in all cases for raising the quantity of all the biochemical constituents in the leaves. The combined influence of the YE and GA_3_ concentrations had a significant effect on the total soluble sugars, total phenolic, total flavonoids, and total glycosides in the leaves. Furthermore, the highest values of all the biochemical constituents in the leaves were detected by utilizing YE at 1.5 g/L with GA_3_ at 300 ppm, as compared to the control, which produced the lowest values. The above-mentioned biochemical constituents were increased by 157.14%, 111.76%, 400% and 308.33%, respectively.

**Figure 5:**
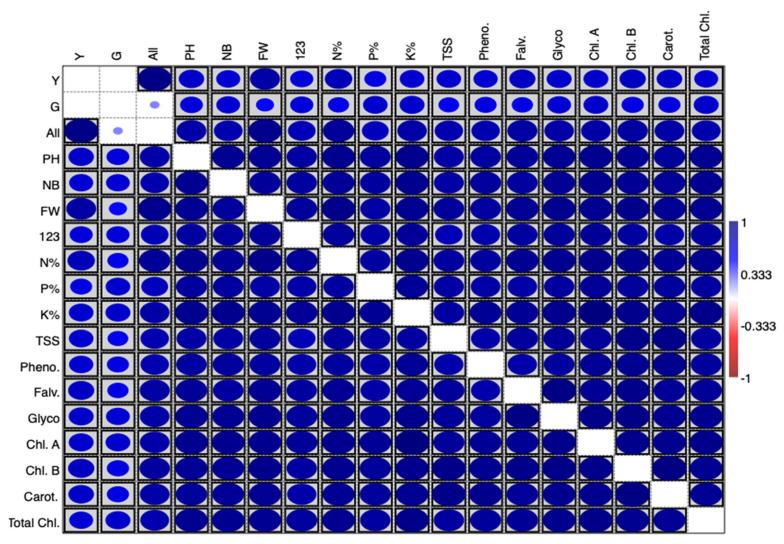
Correlation matrix plot showing the interrelationship between studied variables. The blue colour indicates a positive correlation, and red a negative correlation. Boxes indicate a significant correlation.

A correlation matrix plot showed the interrelationships between the studied variables. The blue colour indicates a positive correlation and red a negative correlation. Boxes indicate a significant correlation (Figure 5). It shows a positive strong significant correlation between treatments, morphological, physiological, and biochemical parameters.

**Figure 6:**
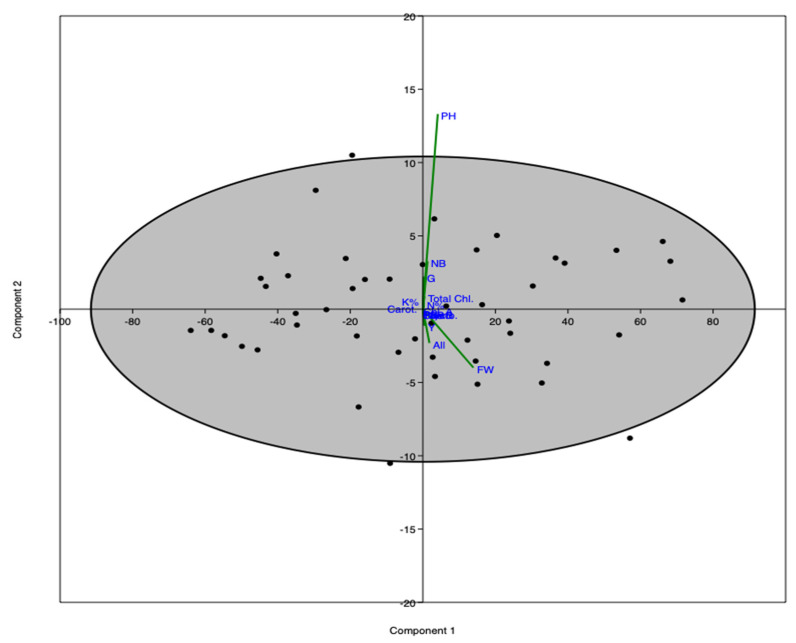
PCA-ordination showing the interrelationship between variables of the study.

PCA-ordination shows the interrelationship between the variables of the study. PCA-1 and PCA-2 represent more than 99% of the total variance of the study (Figure 6). The PCA indicates that various morphological and physiological parameters were most affected by the treatments (YE, GA).

## 4. Discussion

The alkalization of soil has become a global environmental problem and is an important factor limiting agricultural productivity [44]. Using biological safety compounds to improve plant productivity and quality has recently received much attention. Bio-stimulants improve plant growth and development by enhancing photosynthesis, endogenous hormones, ion uptake, nucleic acid, and protein synthesis, among other metabolic activities [45]. In the current study, spraying Solidago plants with yeast extract (YE) and gibberellic acid (GA_3_) individually or in combination markedly enhanced the morphological parameters, physiological responses, macronutrients and biochemical determinations compared to the non-treated plants.

The Increment in growth traits as a result of YE might be due to the presence of different macro and micronutrients, growth regulators, proteins, and vitamins (especially vitamin B) that encourage the plant to produce dry matter [46]. It is also a natural source of cytokinins, which promote cell proliferation and differentiation while also governing shoot and root morphogenesis, chloroplast maturation, protein and nucleic acid synthesis [47], or may be due to YE being high in tryptophan, which is a precursor to indole acetic acid (IAA). This substance promotes cell division and elongation [48]. In addition, the increment might be due to the various roles of amino acids in the protein structure of several plant enzymes that are required for vegetative development [49]. The improvement of physiological properties in response to the foliar application of YE may be attributed to its bio-regulator role in plants, affecting the balance of photosynthesis and photorespiration [50] and delaying the leaf senescence by reducing the degradation of chlorophyll, improving protein and RNA synthesis [51]. The importance of YE at different concentrations on the accumulation of the N, P and K % in leaves may be due to its diverse range of amino acids and vitamins. In addition, YE is a natural source of many growth components as a protective agent, as well as the majority of nutritional elements (Na, Ca, Fe, K, P, S, Mg, Zn, and Si), cytokinins, and several organic compounds [23]. Furthermore, the positive effect of YE on promoting vegetative growth could explain why the concentration of nutritional elements in leaves is increasing [52]. YE stimulated the production of endogenous hormones, which led to the accumulation of secondary metabolites such as total soluble sugars, phenolic, flavonoids, and glycosides [53]. YE plays a major role in increasing carbon dioxide release through the fermentation process, which results in an increase in photosynthetic pigments and successfully activates the photosynthesis process. Alternatively, this might have been due to it promoting cell division and cell elongation, resulting in an increased leaf area [54] as a result of the biosynthesis of carbohydrates being accelerated [55]. Similar reports were earlier published by [56] on *flax* plants [52], wheat plants [22], Chinese carnation, and [24] white lupine plants.

On the other hand, plants sprayed with GA_3_ also caused an increase in plant growth traits. This impact could be explained by gibberellic acid’s ability to boost auxin levels, resulting in increased cell division and elongation [57]. Furthermore, the mechanism involves the hydrolysis of starch as a result of the generation of GA_3_-induced α- amylase, which could raise the concentration of sugars in the cell sap, hence, elevating the osmotic pressure; water enters the cell, causing the cell wall to stretch [58]. Moreover, it is quoted that the meristematic region’s increasing size and the fraction of cells undergoing division [59] contribute to cell elongation and vigorous growth. Foliar application with GA_3_ also caused an increase in pigments, i.e., chlorophyll a, b, and total carotenoids. This effect may be due to GA_3_’s effective role in preventing chloroplast and chlorophyll degradation, resulting in a reduction in leaf senescence and yellowing due to increased chlorophyll synthesis and chloroplast development [57]. Furthermore, under the impact of GA_3_, GA_3_ in the chloroplast membrane enables photosynthesis to be regulated, more light to be trapped, a larger leaf surface, and increased leaf longevity [60]. It is worth mentioning that the increment of essential minerals (N, P, and K) by increasing the concentration of GA_3_ may be a result of augmenting the leaves’ dry weights to a greater extent than its effect on reducing N, P, and K percentages in the leaves [61]. Therefore, the increment in total soluble sugars, total phenolic, total flavonoids and total glycosides as a result of GA_3_ application might be due to the role in increasing the amount of chlorophyll in the leaves, which was reflected in raising the photosynthetic rate and, consequently, the accumulation of secondary metabolites increased [62]. Otherwise, it is likely that increased photosynthetic CO_2_ fixation may increase the amount of carbohydrates available for metabolism and export them to the sink [63]. In harmony with these results were those emphasized by [64] on *chrysanthemums* [65], tuberose plants, and [1] golden rod plants.

**Figure 7:**
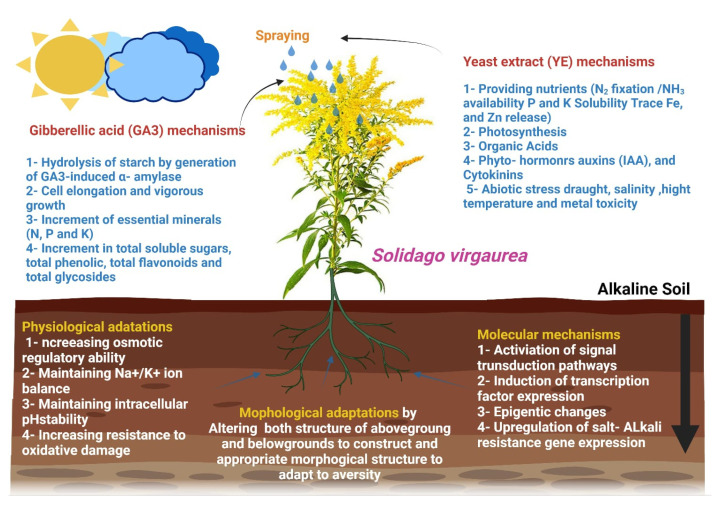
Summary graphical of the suggested mechanism for the effect of yeast extract and gibberellic acid in alkalinity stress tolerance.

Soil osmotic pressure is increased during saline-alkali stress due to sodium ion build-up. To sustain intracellular water potential, plant cells generate and store proline, soluble proteins, betaine, sugar, polyols, and polyamines [66]. These compounds modify water’s solvent characteristics, stabilize the internal osmotic potential, increase protein folding stability, and safeguard the macromolecular structure [37]. Sorghum seedlings respond to salt-alkali conditions by synthesizing proline and soluble proteins. Wheat responded to salt and alkali stress by increasing its proline, soluble sugar, and polyol (sorbitol) content. Furthermore, under saline-alkali stress, many plants release considerable quantities of organic acids, which may buffer intracellular pH and ion balance [66]. Proton pump H+-ATPase may have a role in organic acid release from roots under NaHCO_3_ stress, according to related research [38]. Finally, saline-alkali stress causes osmotic and ionic stress, which generates reactive oxygen species (ROS) such as H_2_O_2_ and hydroxyl radicals [39]. Alkali stress damaged rice cell membranes, which boosted the plant’s antioxidant defence mechanism [40]. Taken together, our findings demonstrate that plants of various species and cultivars within the same species may alter their osmotic adjustment components in response to salt-alkali stress, as shown in Figure 7.

## 5. Conclusions

The earlier findings demonstrate that yeast extract (YE) and gibberellic acid (GA_3_) are crucial for Solidago plants. Likewise, YE and GA_3_ concentrations proved to have remarkably positive effects on morpho-physiological parameters, macronutrients, and biochemical determinations. Thus, it can be recommended that spraying YE alone principally at the concentration of 1.5 g/L or in combination with GA_3_, especially at the concentration of 300 ppm, has the possibility of enhancing and progressing the quantity and quality characteristics of Solidago plants. This treatment significantly increased the plant growth, photosynthetic efficiency, macronutrient content, and biochemical constituents. Its application may be profitable for farmers and producers, particularly in the world’s arid and semi-arid regions.

## Figures and Tables

**Table 1:** Some initial physio-chemical properties of the studied soil.

Properties	Value	
	2018	2019
Particle size distribution		
Coarse sand %	7.40	7.44
Fine sand %	41.61	41.80
Silt %	18.17	18.23
Clay %	32.82	32.80
Texture class	Sandy clay loam	
PH	7.82	7.81
ECe (dS/m)	3.42	3.40
Soluble cations (meq 100/g soil)		
Ca^2+^	7.92	7.94
Mg^2+^	6.80	6.90
Na^+^	15.27	15.33
K^+^	0.48	0.45
Soluble anions (meq /100 g soil)		
Cl^−^	15.98	15.95
HCO_3_^−^	0.86	0.85
SO_4_^2−^	17.27	17.25
CO_3_^2−^	0.00	0.00
Nutrients available		
N%	0.18	0.20
P%	0.22	0.24
K%	0.32	0.30

**Table 2:** Major components of yeast extract.

Amino Acid %		Vitamins (mg/100 g DW)		Minerals	
Alanine	1.69	Vit.B1	23.33	Nitrogen	6.88%
Arginine	1.49	Vit.B2	21.04	Phosphorus	0.66%
Aspartic acid	2.32	Vit.B6	20.67	potassium	0.95%
Cystine	0.63	Vit.B12	19.17	Magnesium	0.19%
Glutamic acid	3.76	Thiamine	23.21	Calcium	0.17%
Glycine	1.45	Riboflavin	27.29	Sulphur	0.48%
Histidine	0.71	Inositol	20.43	Iron	107 ppm
Isoleucine	0.85	Biotin	20.04	Zinc	77 ppm
Leucine	1.91	Nicotinic acid	73.92	Copper	5 ppm
Lysine	1.13	Pantothenic acid	38.43	Manganese	13 ppm
Phenylalanine	1.18	P aminobenzoic acid	29.49	**Growth regulators (ppm)**	
Proline	1.29	Folic acid	26.22	Adenine	31
Serine	1.98	Pyridoxine	22.09	Betaines	56
Threonine	1.54			**Others %**	
Tryptophan	0.25			Crude Protein	43.00
Tyrosine	0.99			Crude Fat	2.20
Valine	1.4			Carbohydrates	33.21
Methionine	0.4			Crude Fibre	7.20
				Ash	3.80

Cited from [33].

**Table 3:** The effect of yeast extract and gibberellic acid on the morphological measurements of *Solidago virgaurea* plants in terms of plant height, no. of branches, and shoot FW and DW.

Treatments	Plant Height (cm)	No. of Branches	Weight of Shoot System (g Plant^−1^)	
			FW	DW
YE (g/L)				
0.0 (Control)	56.5 ± 2.9 d	9.5 ± 0.8 d	88.7 ± 4.1 d	58.4 ± 3.9 d
0.5	64.3 ± 3.4 c	11.8 ± 0.8 c	114.3 ± 4.3 c	67.5 ± 4.0 c
1.0	72.0 ± 3.0 b	13.4 ± 0.8 b	137.9 ± 4.1 b	99.2 ± 4.4 b
1.5	80.2 ± 2.6 a	15.6 ± 0.8 a	166.6 ± 4.4 a	107.0 ± 3.8 a
ANOVA-1-way	<0.001 ***	<0.001 ***	<0.001 ***	<0.001 ***
GA (ppm)				
0.0 (Control)	58.4 ± 3.4 d	9.5 ± 0.9 d	104.9 ± 4.3 d	70.5 ± 4.4 d
100	64.4 ± 2.9 c	11.4 ± 0.7 c	119.8 ± 3.9 c	78.2 ± 3.4 c
200	71.8 ± 2.9 b	13.8 ± 0.8 b	134.7 ± 4.1 b	87.6 ± 4.2 b
300	78.4 ± 2.8 a	15.5 ± 0.8 a	148.0 ± 4.5 a	95.9 ± 4.1 a
ANOVA-1-way	<0.001 ***	<0.001 ***	<0.001 ***	<0.001 ***
Two-way analysis of variance				
Corr. model	<0.001 ***	<0.001 ***	<0.001 ***	<0.001 ***
YE	<0.001 ***	<0.001 ***	<0.001 ***	<0.001 ***
GA	<0.001 ***	<0.001 ***	<0.001 ***	<0.001 ***

*** indicate differences at ≤ 0.001 probability level. Means (±SE) followed by different letters in the columns are significantly different according to DMRTs.

**Table 4:** The effect of different YE and GA3 concentrations on the chl-a, chl-b, and carotenoids levels of *Solidago virgaurea* plants.

Treatments	Chl. a	Chl. b	Total Carotenoids
	mg mm^−2^		
YE (g/L)			
0.0 (Control)	1.80 ± 0.04 d	0.48 ± 0.02 d	0.39 ± 0.02 d
0.5	2.02 ± 0.04 c	0.57 ± 0.02 c	0.46 ± 0.02 c
1.0	2.25 ± 0.03 b	0.67 ± 0.02 b	0.52 ± 0.02 b
1.5	2.46 ± 0.03 a	0.80 ± 0.03 a	0.60 ± 0.03 a
ANOVA-1-way	<0.001 ***	<0.001 ***	<0.001 ***
GA (ppm)			
0.0 (Control)	1.82 ± 0.04 d	0.49 ± 0.02 d	0.40 ± 0.02 d
100	2.00 ± 0.03 c	0.59 ± 0.03 c	0.46 ± 0.03 c
200	2.25 ± 0.04 b	0.67 ± 0.02 b	0.52 ± 0.02 b
300	2.47 ± 0.04 a	0.76 ± 0.02 a	0.58 ± 0.02 a
ANOVA-1-way	<0.001 ***	<0.001 ***	<0.001 ***
Two-way analysis of variance			
Corr. model	<0.001 ***	<0.001 ***	<0.001 ***
YE	<0.001 ***	<0.001 ***	<0.001 ***
GA	<0.001 ***	<0.001 ***	<0.001 ***

*** indicate differences at *p* ≤ 0.001 probability level. Means (±SE) followed by different letters in the columns are significantly different according to DMRTs.

**Table 5:** The effect of yeast extract and gibberellic acid on the macronutrients constitute of (*Solidago virgaurea*) plants.

Treatments	N	P	K
	%		
YE (g/L)	*	*	*
0.0 (Control)	2.87 ± 0.06 d	0.35 ± 0.03 d	3.42 ± 0.08 d
0.5	3.35 ± 0.07 c	0.42 ± 0.03 c	3.84 ± 0.08 c
1.0	3.64 ± 0.07 b	0.48 ± 0.03 b	4.25 ± 0.06 b
1.5	4.18 ± 0.07 a	0.54 ± 0.03 a	4.64 ± 0.06 a
ANOVA-1-way	<0.001 ***	<0.001 ***	<0.001 ***
0.0 (Control)	3.06 ± 0.07 d	0.33 ± 0.03 d	3.45 ± 0.07 d
100	3.32 ± 0.06 c	0.42 ± 0.04 c	3.78 ± 0.06 c
200	3.64 ± 0.08 b	0.49 ± 0.03 b	4.25 ± 0.07 b
300	4.01 ± 0.07 a	0.55 ± 0.03 a	4.66 ± 0.07 a
ANOVA-1-way	<0.001 ***	<0.001 ***	<0.001 ***
Two-way analysis of variance			
Corr. model	<0.001 ***	<0.001 ***	<0.001 ***
YE	<0.001 ***	<0.001 ***	<0.001 ***
GA	<0.001 ***	<0.001 ***	<0.001 ***

*, *** indicate differences at *p* ≤ 0.05, ≤ 0.001 probability level. Means (±SE) followed by different letters in the columns are significantly different according to DMRTs.

**Table 6:** The effect of yeast extract and gibberellic acid on the macronutrients constitute of (*Solidago virgaurea*) plants.

Treatments	Total Soluble Sugars	Total Phenolic	Total Flavonoids	Total Glycosides
	mg/g DW			
YE (g/L)				
0.0 (Control)	0.18 ± 0.01 d	0.21 ± 0.01 d	0.09 ± 0.01 d	0.20 ± 0.01 d
0.5	0.21 ± 0.02 c	0.25 ± 0.01 c	0.11 ± 0.01 c	0.25 ± 0.01 c
1.0	0.25 ± 0.01 b	0.27 ± 0.02 b	0.15 ± 0.01 b	0.31 ± 0.02 b
1.5	0.29 ± 0.02 a	0.31 ± 0.02 a	0.20 ± 0.01 a	0.38 ± 0.01 a
ANOVA-1-way	<0.001 ***	<0.001 ***	<0.001 ***	<0.001 ***
GA (ppm)	*	*	*	*
0.0 (Control)	0.19 ± 0.01 d	0.22 ± 0.01 d	0.09 ± 0.01 d	0.20 ± 0.02 d
100	0.22 ± 0.02 c	0.24 ± 0.01 c	0.12 ± 0.01 c	0.26 ± 0.01 c
200	0.25 ± 0.02 b	0.27 ± 0.01 b	0.15 ± 0.01 b	0.32 ± 0.01 b
300	0.28 ± 0.02 a	0.30 ± 0.01 a	0.18 ± 0.01 a	0.37 ± 0.01 a
ANOVA-1-way	<0.001 ***	<0.001 ***	<0.001 ***	<0.001 ***
Two-way analysis of variance				
Corr. model	<0.001 ***	<0.001 ***	<0.001 ***	<0.001 ***
YE	<0.001 ***	<0.001 ***	<0.001 ***	<0.001 ***
GA	<0.001 ***	<0.001 ***	<0.001 ***	<0.001 ***

*, *** indicate differences at *p* ≤ 0.05, ≤ 0.001 probability level. Means (±SE) followed by different letters in the columns are significantly different according to DMRTs.
